# Respiratory tract clinical sample selection for microbiota analysis in patients with pulmonary tuberculosis

**DOI:** 10.1186/2049-2618-2-29

**Published:** 2014-08-25

**Authors:** Luz Elena Botero, Luisa Delgado-Serrano, Martha Lucía Cepeda, Jose Ricardo Bustos, Juan Manuel Anzola, Patricia Del Portillo, Jaime Robledo, María Mercedes Zambrano

**Affiliations:** 1 Molecular Genetics & Biotechnology, Corporación CorpoGen, Carrera 5 No. 66A-34, Bogotá, DC 110231, Colombia; 2 Unidad de Bacteriología y Micobacterias, Corporación para Investigaciones Biológicas, Medellín 050034, Colombia; 3 Facultad de Medicina, Escuela de Ciencias de la Salud, Universidad Pontificia Bolivariana, Medellín 050031, Colombia

**Keywords:** Microbiota, Respiratory tract, Pulmonary tuberculosis, ITS1, 16S rRNA, Microbial diversity, *Mycobacterium tuberculosis*

## Abstract

**Background:**

Changes in respiratory tract microbiota have been associated with diseases such as tuberculosis, a global public health problem that affects millions of people each year. This pilot study was carried out using sputum, oropharynx, and nasal respiratory tract samples collected from patients with pulmonary tuberculosis and healthy control individuals, in order to compare sample types and their usefulness in assessing changes in bacterial and fungal communities.

**Findings:**

Most V1-V2 16S rRNA gene sequences belonged to the phyla Firmicutes, Bacteroidetes, Proteobacteria, Actinobacteria, and Fusobacteria, with differences in relative abundances and in specific taxa associated with each sample type. Most fungal ITS1 sequences were classified as Ascomycota and Basidiomycota, but abundances differed for the different samples. Bacterial and fungal community structures in oropharynx and sputum samples were similar to one another, as indicated by several beta diversity analyses, and both differed from nasal samples. The only difference between patient and control microbiota was found in oropharynx samples for both bacteria and fungi. Bacterial diversity was greater in sputum samples, while fungal diversity was greater in nasal samples.

**Conclusions:**

Respiratory tract microbial communities were similar in terms of the major phyla identified, yet they varied in terms of relative abundances and diversity indexes. Oropharynx communities varied with respect to health status and resembled those in sputum samples, which are collected from tuberculosis patients only due to the difficulty in obtaining sputum from healthy individuals, suggesting that oropharynx samples can be used to analyze community structure alterations associated with tuberculosis.

## Findings

Recent studies suggest that microbial communities inhabiting the human body can influence the host's health status and contribute to disease [1]. The human upper respiratory tract represents the major portal of entry for numerous airborne microorganisms, such as bacteria, fungi, or viruses [2]. High-throughput sequencing methods have provided great insight regarding the composition of the respiratory tract-associated microbiota, which has been recently related with the development of diseases such as asthma [3], nosocomial pneumonia, pulmonary cystic fibrosis [4], and chronic obstructive pulmonary disease [5].

Tuberculosis (TB), a respiratory disease caused by *Mycobacterium tuberculosis* (*Mtb*), is a major global public health problem that affects millions of people each year and ranks as the second leading cause of death from an infectious disease worldwide, with 8.6 million new cases and 1.3 million deaths in 2012 (25% of them were HIV-associated) [6]. The *Mtb* pathogen typically affects the lungs (pulmonary TB) but can affect other sites as well (extrapulmonary TB). Individuals with pulmonary TB can expel bacteria by talking, coughing, or sneezing, spreading the pathogen through airborne particles that are inhaled by others. The complex *Mtb*-human host interaction and the resulting infectious process indicate that TB disease development may be a multifactorial process [7]. Microorganism characteristics coupled to local host immune response determine whether bacilli are cleared or will lead to either acute or latent disease [2].

Recent studies of the respiratory tract microbiota using sputum samples and mixtures of saliva and pharyngeal secretions indicate changes and possible associations with pulmonary TB [8,9]. In this work, we examined the microbiota in three types of respiratory tract samples, nasal and oropharynx swabs and sputum, the latter taken only from patients since sputum is difficult to procure from healthy individuals, not to mention the more invasive bronchoalveolar lavage. Previous studies have shown that oropharyngeal swabs can be a reasonable proxy for lung samples [10], and an analysis in healthy individuals indicated that lung and upper airway bacterial populations, which include the oropharynx, were largely indistinguishable from one another [11]. Given that the resemblance between oropharyngeal and sputum communities is still unclear and the difficulty of getting sputum samples from healthy individuals, the aim of this work was to use different sample types and determine which one could be used to evaluate the composition of the respiratory tract microbiota associated with TB patients and healthy controls.

### Population and sampling

To assess respiratory tract microbiota associated with TB patients and healthy controls, we collected nasal, oropharynx, and sputum samples from six TB patients and nasal and oropharynx samples from six healthy controls. The inclusion and exclusion criteria can be found in Additional file 1, and the demographic and clinical characteristics of individuals are shown in Additional file 2. Nasal samples were taken by swabbing the mucosal surface of the deep nasal cavity by doing ten rotational movements in each nostril; oropharynx swabs were taken from the back wall of the oropharynx, avoiding contact with other surfaces such as tonsil, palate, and tongue. As previously reported, the median body mass index (BMI) was significantly lower in TB patients (19.6) compared to healthy controls (25.5) (Table 1) [12]. All sputum, nasal and oropharynx samples were collected, processed as reported [13], and used to isolate DNA with the MoBio PowerSoil DNA Isolation Kit (MO Bio Laboratories, Carlsbad, CA, USA) [14,15], following the manufacturer's recommendations.

**Table 1:** Population characteristics

	**TB patients**	**Controls**
Number	6	6
Age; median (range) in years	38 (30–46)	37 (26–47)
Gender: male/female	5/1	5/1
Body mass index (BMI; kg/m^2^) median (max/min)*	19.1 (21.2–16.4)	25.5 (27.6–22.5)

*p < 0.05 (obtained with *U* Mann-Whitney). Significant difference between TB patients and healthy individuals.

### Bacterial diversity

**Figure 1:**
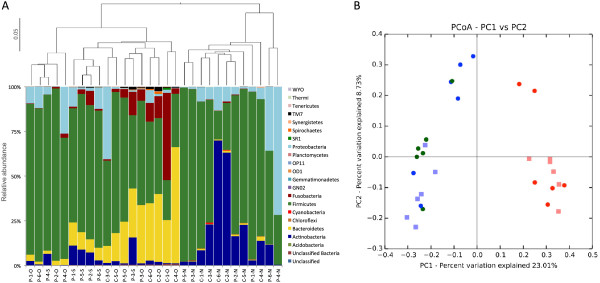
**Analysis of bacterial 16S rRNA gene sequences. (A)** Taxonomic classification (*bottom*) and UPGMA analysis based on unweighted UniFrac metric (*top*) for sequences obtained from TB patient (*P*) or healthy control (*C*) sputum (*S*), oropharynx (*O*), and nasal (*N*) samples. Different individuals are indicated by *numbers*. **(B)** PCoA UniFrac weighted analysis of sputum (*green*), oropharynx (*blue*), and nasal (*red*) samples for controls (*squares*) and patients (*circles*).

The V1-V2 hypervariable region of the bacterial 16S rDNA was amplified with primers 27F (5′ AGAGTTTGATCCTGGCTCAG 3′) and 338R (5′ TGCTGCCTCCCGTAGGAGT 3′) [16], using 10 ng DNA and AccuPrime™ Taq DNA polymerase (Invitrogen, Thermo Fisher Scientific, Waltham, MA, USA) with the following conditions: 95°C for 3 min, followed by 35 cycles of 20 s at 95°C, 20 s at 52°C and 60 s at 65°C, and ending with 6 min at 72°C. Samples were sequenced using 454/Roche GS-FLX Titanium chemistry (EnGenCore, University of South Carolina, Columbia, SC, USA). Pyrosequencing reads have been submitted to the NCBI Sequence Read Archive (BioProject no. PRJNA242354). All sequence analyses were carried out using Quantitative Insights Into Microbial Ecology (QIIME) v1.6 [17]. Approximately 589,000 sequences with a length size larger than 200 bps remained after quality filtering (386,645 and 202,422 reads from TB patient and control samples, respectively) using a quality score of 25 with a slide window of 40 bases. The open-reference operational taxonomic unit (OTU) picking protocol was used to discard sequences that were likely not rRNA and chimeras using 97% sequence identity and the Greengenes core set [18]. Samples were rarified to the minimum number of sequence reads per sample (the number varied from 10,480 to 38,099), and taxonomic classification was performed using the Ribosomal Database Project naïve Bayesian classifier [19]. Chao1 and Shannon indexes were calculated for taxon richness and diversity estimations, respectively. Significance tests were performed using the non-parametric Mann-Whitney *U* test (SPSS V.18, SPSS Inc, Chicago, IL, USA). A first comparison showed that sputum samples had the highest diversity, followed by oropharynx and the least diverse were nasal samples. Both nasal and oropharynx samples from healthy controls were more diverse than samples from TB patients, with a significant difference in the Shannon index for nasal samples (Table 2). Most sequences in all samples (>99% in TB patients and >98% in healthy controls) belonged to five phyla, Firmicutes, Bacteroidetes, Proteobacteria, Actinobacteria, and Fusobacteria, consistent with previous reports [9,20,21] (see Figure 1A). White's non-parametric *t* test (pairwise comparisons) [22], ANOVA (multiple comparisons), and false discovery rate (FDR) correction, all implemented in the STAMP software [23], were used to identify groups that could be characteristic of each sample type. STAMP results showed that of the predominant phyla, only Bacteroidetes (*p* = 0.017) and Thermi (*p* = 0.020) were significantly different among sample types (nasal, oropharynx, and sputum). Principal coordinate analyses (PCoA) and unweighted pair group method with arithmetic mean (UPGMA) analyses performed to compare communities indicated that oropharynx and sputum microbial communities clustered together, whereas nasal samples clustered separately, consistent with previous analyses of oropharynx and nasal communities [14,20] (Figure 1). Between-group versus within-group UniFrac distances, with permutation, were analyzed using Student's *t* test for significant differences of averages to see if communities from the same sample type were more similar to one another than to the other communities. The oropharynx sample communities were as similar to the sputum sample communities as they were to each other (*p* > 0.05, data not shown), and likewise, communities from sputum samples were also indistinguishable from oropharynx communities, indicating that they are closely related.

**Table 2:** Sequence data and diversity indexes

**Sample type**	**Characteristics**	**Sequence type**			
		**Bacterial (16S)**		**Fungal (ITS1)**	
		**TB**	**Controls**	**TB**	**Controls**
**Nasal**	Total sequences^a^	124,977	106,729	76,480	73,858 (4)
	Observed OTUs	318	348	378	345
	Chao1	708	627	684	585
	Shannon (H')	3.0*	4.2*	6.9	6.6
**Oropharynx**	Total sequences^a^	140,987	95,693	29,759	22,376 (3)
	Observed OTUs	577	882	98	131
	Chao1	1262	1941	153	209
	Shannon (H')	4.8	5.8	3.8	4.8
**Sputum**	Total sequences	120,681	ND	66,278	ND
	Observed OTUs	827	ND	102	ND
	Chao1	1857	ND	154	ND
	Shannon (H')	6.1	ND	3.4	ND

*ND* not available. ^a^Number in parenthesis indicates samples for which sequences were obtained, if less than 6. *p < 0.05 (*U* Mann-Whitney) indicates significance between TB patients and healthy controls for 16S rRNA gene sequences.

These differences were marked by a higher abundance of some phyla, particularly Bacteroidetes and Fusobacteria in oropharynx samples and Thermi in nasal swabs (*p* values = 0.034, 0.030, and 0.031, respectively). Fourteen taxa differed significantly between nasal and oropharynx samples when both patient and control groups were analyzed together, but only some of these showed differences within each group: one for patients versus three phyla for controls (Table 3). When comparing sputum and oropharynx communities, only for TB patients for which both samples were collected, the only observed difference was in Actinobacteria, which was significantly higher in sputum samples (Figure 1A, Table 3); no significant differences were found at other phylogenetic levels. As expected, sequences belonging to the genus *Mycobacterium* were detected only in sputum but not in patient oropharynx samples, consistent with culture results.

**Table 3:** Phyla that differ significantly between sample types

**Phylum**	**Mean relative abundance**					**Sample comparison**				
	**TB patients**			**Controls**		**O vs N**			**O vs S**	**N vs S**
	**Sputum**	**Oropharynx**	**Nasal**	**Oropharynx**	**Nasal**	**All samples**	**Controls**	**TB patients**	**TB patients**	**TB patients**
**Bacteria**										
Bacteroidetes	11.015	11.16	0.30	30.72	1.02	0.00366	0.0095			
Cyanobacteria	0	0.0016	0.064	0	0.22	0.00314				
TM7	0.48	0.19	0.011	0.82	0.024	0.00799				
Fusobacteria	4.30	2.49	0.10	12.84	0.26	0.0044				
Thermi	0	0	0.081	0	0.062	0.00275		0.013		0.034
Actinobacteria	8.52	0.81	5.91	1.41	33.49	0.022	0.018		0.013	
Unclassified Bacteria	0.25	0.21	0.064	0.48	0.043	0.011	0.0095			0.049
Spirochaetes	0.070	0.071	0	0.30	0	0.00549				
SR1	0.0016	0.0079	0	0.18	0	0.00733				
Gemmatimonadetes	0	0	0.016	0	0	0.00477				
Chloroflexi	0	0	0.0048	0	0.036	0.0044				
Acidobacteria	0	0	0.011	0	0	0.029				
Tenericutes	0.0016	0.0079	0.0016	0.016	0	0.03				
**Fungi**										
Ascomycota	76.89	74.42	45.24	43.30	23.89	0.036		0.009		
Unclassified Fungi	0.087	1.4	0.024	10.32	0			0.011		0.018

The relative abundance of the various phyla is shown on the left side. *p* values are shown on the right, only for phyla that were significantly different when comparing between sample types from patients and controls together (all samples), or the control and TB patient groups separately. *p* values were corrected using FDR. *O* oropharynx, *N* nasal, *S* sputum.

Samples were also analyzed in order to see changes in respiratory tract bacterial communities associated to health status. The only difference between patient and control groups, using either nasal and oropharynx samples separately or both sample types (nasal and oropharynx) together, was found in oropharynx samples, where unclassified sequences belonging to the Streptococcaceae family were more abundant in TB patients (*p* = 0.00878, not shown). Taken together, these observations indicate alterations in these communities and raise the possibility that such imbalances could affect, or result from, infection and/or colonization.

### Fungal diversity

**Figure 2:**
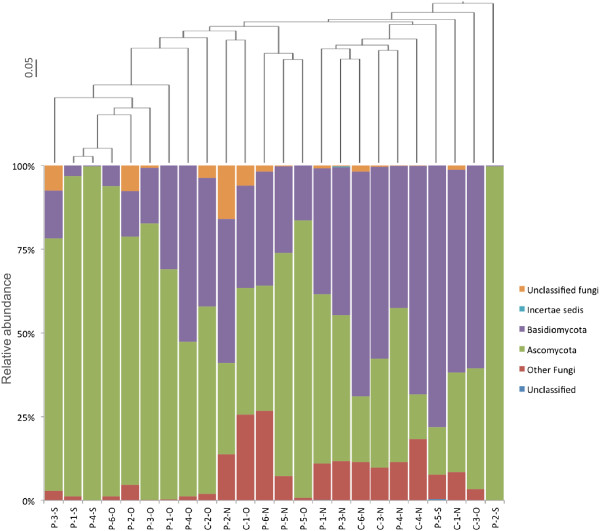
**Phylum level analysis of fungal ITS1 sequences.** The *bottom* shows classification for sequences obtained from TB patient (*P*) and healthy control (*C*) sputum (*S*), oropharynx (*O*), and nasal (*N*) samples. The *top* indicates clustering analysis based on Jaccard distances.

The fungal nuclear ribosomal internal transcribed spacer ITS1 region was amplified using the primer set ITS-5 (5′GGAAGTAAAAGTCGTAACAAGG3′) and ITS-2 (5′GCTGCGTTCTTCATCGATGC3′) [24] and conditions as indicated above for Bacteria, but doing 35 cycles of 60 s at 94°C, 60 s at 55.2°C, and 90 s at 72°C, followed by a final extension for 10 min at 72°C. Amplicons were subjected to pyrosequencing, and sequence analysis was done as indicated above for Bacteria. Of a total of 783,925 raw sequences obtained, 268,751 sequences with a length size larger than 100 bps were retained after filtering for quality (34.3%). Chimeras and non-rRNAs sequences were discarded, as mentioned above for Bacteria, using 97% sequence identity set of fungal ITS sequences from the UNITE database [25]. Samples were rarified to 2,076 reads per sample (the number of reads per sample ranged from 1 to 42,479), leaving only 17 samples from patients (out of 18) and 7 from controls (out of 12). Nasal samples showed greater fungal richness and diversity, although the differences between patients and controls in samples of the same type were not significant (Table 2). Overall, the majority of the ITS1 sequences analyzed (90%) were classified as belonging to the phylum Ascomycota, followed by Basidiomycota. This was observed for all sample types with the exception of nasal samples from healthy control individuals (Figure 2), and is consistent with nasal fungal analysis in the nares [26]. However, the genus *Malassezia* was not predominant in this study, as has been reported previously for diverse skin sites, probably due to different environmental conditions of the body sites sampled [26]. Again, communities clustered according to sample type (oropharynx, nasal, and sputum) (Figure 2), and TB patient sputum and oropharynx samples showed similar relative abundances with no significant differences at the phylum level (Figure 2, Table 3). Significant differences were observed only when comparing patient nasal communities with those of the oropharynx (Ascomycota and unclassified sequences) or sputum (unclassified sequences) (Table 3). Similar to Bacteria, differences between patients and controls were observed only in oropharynx samples, with a decrease of the genus *Cryptococcus* in patients (*p* = <1e-15, not shown). In TB patients, *Candida* and *Aspergillus* were the most frequent genera for both sputum and oropharyngeal samples, even though no significant differences were found when compared with healthy controls. In contrast to Bacteria, significant differences at the phylum level between oropharynx and nasal sample communities were seen only in patients with TB but not in controls (Table 3). Previous work on skin microbial communities indicated that bacterial and fungal richness did not show a linear correlation and that diversity was dependent on body site [26]. Similarly, in this study, the diversity of bacterial and fungal communities was found to vary inversely between samples analyzed: bacterial diversity was greater in oropharynx when compared with nasal samples, whereas fungi were more diverse in nasal than in oropharynx samples (Table 2).

### Conclusions

Differences in community diversity indexes and in abundance of particular taxa, specifically in oropharynx communities, between TB patients and healthy controls suggest disturbance of respiratory tract microbial communities, despite the overall similarity in terms of the major phyla identified. These altered communities could either result from or influence infection and/or colonization by *M. tuberculosis*, a possibility that can be further examined by studying changes in particular taxa or in functionality via metagenomic sequencing using samples collected at various time points. More importantly, there was a resemblance between communities from sputum in TB patients and those present in the oropharynx, both of which were distinct from the nasal microbiota. This study therefore indicates that oropharynx samples can be valuable for probing respiratory tract microbiota and sets the groundwork for more extensive comparison and analysis of possible microbial community imbalances associated with a diseased state such as TB.

### Ethics statement

The research complied with the standards and recommendations for biomedical research involving human subjects adopted by the 18th World Medical Assembly, Helsinki, Finland, June 1964 and the 59th Meeting, Seoul, 2008. Ethical standards also complied with resolution N°008430 (1993) established by the Colombian Ministry of Health for work with humans. Informed written consent was obtained from all participants prior to enrollment with approval by the Ethics Committee of Corporación Corpogen (Bogotá), Corporación para Investigaciones Biológicas-CIB (Medellín) and with the approval of the Research Committee METROSALUD, ESE (Medellín).

## Abbreviations

*BMI*: Body mass index; *HIV*: Human immunodeficiency virus; *ITS1*: Internal transcribed spacer region 1; *Mtb*: *Mycobacterium tuberculosis*; *OTU*: Operational taxonomic unit; *PCoA*: Principal coordinate analyses; *QIIME*: Quantitative Insights Into Microbial Ecology; *TB*: Tuberculosis; *UPGMA*: Unweighted pair group method with arithmetic mean.

## Competing interests

The authors declare that they have no competing interests.

## Authors' contributions

PDP, MMZ, JR, and LEB were involved in experimental design. LEB assembled the population and epidemiological data and performed the sampling. LEB and MLC were involved in the molecular procedures. LDS, JRB, and JMA developed workflows for the analysis of bacterial and fungal diversity. LEB, LDS, MLC, JMA, and MMZ analyzed the data. LEB, LDS, MLC, and MMZ wrote and edited the paper. All authors read and approved the final manuscript.

## Supplementary Material

Additional file 1 **Definition of groups and inclusion and exclusion criteria.** Parameters used to select the individuals for the study. Click here for file

Additional file 2 **Demographic and clinical characteristics of the population.** Metadata that describes the most important demographic and clinical features of the study individuals. Click here for file
