# Supplementary material for: Respiratory tract clinical sample selection for microbiota analysis in patients with pulmonary tuberculosis
Source: Microbiome. 2014 Aug 25;2:29. doi: 10.1186/2049-2618-2-29 (PMC4164332; doi:10.1186/2049-2618-2-29)
Supplement: Additional file 1 — Definition of groups and inclusion and exclusion criteria. Parameters used to select the individuals for the study. [file 2049-2618-2-29-S1.pdf]

Additional File 1. Definition of groups and inclusion and exclusion criteria

	Patient with Tuberculosis	Healthy Control
**Definition**	Patients consulting in the city of Medellin with clinical symptoms of tuberculosis and no previous history of the disease, they were smear-positive and were not receiving antibiotic treatment.	People in good health, free of respiratory symptoms, and who had no contact with patients diagnosed with TB.
**Inclusion criteria**	- Persons 18 years of age and older<br>- Either gender (Man or woman)<br>- Diagnosed with TB for the first time<br>- Smear positive acid-fast bacilli<br>- Culture positive for *M. tuberculosis*<br>- Negative serology for HIV	- Persons 18 years of age and older<br>- Either gender (Man or woman)<br>- No prior or current contact with documented TB patient<br>- Negative serology for HIV<br>- Negative response to the PPD test<br>- Matched with TB patients in age (±5 years) and gender
**Exclusion criteria**	- Pregnant women<br>- Previous TB diagnosis at any time<br>- Current or prior treatment for TB<br>- Any type of antibiotic treatment in the last 4 weeks<br>- Diagnosis of cancer<br>- Transplant patients<br>- Diagnosis of cystic fibrosis<br>- Diagnosis of pulmonary disease such as COPD<br>- Diagnosis of autoimmune disease.<br>- Current treatment with immunosuppressive therapies	- Pregnant women<br>- Previous TB diagnosis at any time<br>- Current clinical symptoms suggestive of tuberculosis or any respiratory disease<br>- Prior treatment for TB<br>- Respiratory symptoms or diagnosis of any respiratory diseases other than tuberculosis in the past 4 weeks: tonsillitis, pharyngitis, otitis, epiglottitis, pneumonia, bronchitis, Pneumonitis, COPD.<br>- Any type of antibiotic treatment in the last 4 weeks<br>- Previous or current contact with TB disease patients<br>- Be a health worker in contact with TB patients or suspected of having TB.<br>- Diagnosis of cancer<br>- Transplant patients<br>- Diagnosis of cystic fibrosis<br>- Diagnosis of pulmonary disease such as COPD<br>- Diagnosis of autoimmune disease<br>- Current treatment with immunosuppressive therapies
